# Isolated Axillary Lymph Node Metastasis from Serous Ovarian Cancer

**DOI:** 10.1155/2012/307567

**Published:** 2012-10-24

**Authors:** Hemant Goyal, Vijay K. Mattoo, Umesh Singla

**Affiliations:** ^1^Department of Internal Medicine, Wyckoff Heights Medical Center, 374, Stockholm Street, Brooklyn, NY 11237, USA; ^2^Department of Hematology & Oncology, Wyckoff Heights Medical Center, Brooklyn, NY 11237, USA

## Abstract

A 68-year-old female with past medical history of stage IIIc serous ovarian cancer after cytoreductive surgery and adjuvant chemotherapy came to clinic for regular follow-up visit. Physical examination was completely normal except for an isolated left axillary lymph node enlargement. Patient's abdominal sonogram and CT scan of abdomen and pelvis did not show any other new metastasis. Surgical excisional biopsy of the lymph node was performed and pathology revealed features of metastatic serous ovarian carcinoma.

## 1. Introduction

Ovarian cancer is the most common cause of gynecologic cancer deaths in women in developed countries. This silent killer has high rate of mortality because patients often present to physicians in late stages of the disease and are usually not amenable to curative therapies. The finding of isolated distant metastasis is very unusual after treatment. Moreover, axillary lymph node metastasis from ovarian carcinoma is extremely rare and is considered as a poor prognostic sign. To the best of our knowledge, only one case of single isolated metastasis of ovarian cancer to the axilla has been described previously in medical literature [1].

## 2. Case Report

A 68-year-old female patient with past medical history of stage IIIc serous ovarian carcinoma after cytoreductive surgery and adjuvant chemotherapy with carboplatin and paclitaxel presented to the clinic for regular follow-up visit. Patient denied loss of appetite, weight loss, lethargy, and weakness. On physical examination, a 2 cm diameter, nontender, round, firm, and fixed lymph node was found in left axilla of the patient. Patient was not aware of this mass. Bilateral breast examination was unremarkable.

**Figure 1:**
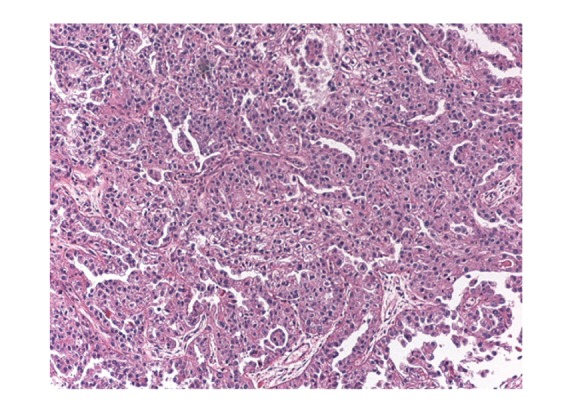
Malignant serous carcinoma of ovary.

**Figure 2:**
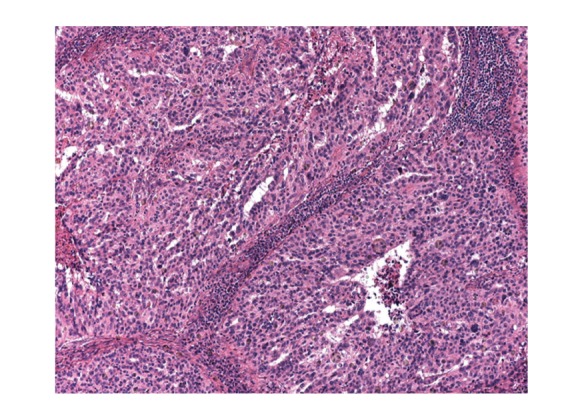
Axillary lymph node metastasis from ovary.

Sonogram of bilateral breasts was performed which showed a 2.9 cm × 2.1 cm left axillary lymph node (ALN) and was negative for any breast mass. Patient CA-125 level was 104 U/mL which was within normal limits prior to this visit (Table 1). CT scan of abdomen and pelvis was negative for any other new metastasis. Excisional biopsy of left ALN was performed and pathology revealed metastatic adenocarcinoma with features consistent with primary ovarian serous carcinoma (Figures 1 and 2). Immunohistochemical staining was positive for WT-1, p16, and p53. It was also positive for CK7 and E-cadherin while CK20 and GCDFP-15 were negative. Patients CA-125 level came down to 5 U/mL 2 months after the excisional biopsy.

## 3. Discussion

Although patients with ovarian carcinoma usually present to physicians with metastasis to other organs but metastasis to ALN is very rare. Ovarian carcinoma primarily metastasizes by directly seeding in to peritoneal cavity. Other common route of spread is lymphatic invasion and transcoelomic spread to adjacent viscera. Our patient had metastasis to left side of colon for which she underwent left hemicolectomy. Retrospective study by Cormio et al. revealed that the most common site of ovarian cancer metastasis is lung which is followed by pleura [2]. Most frequent sites of lymph node involvement are abdominal (47%), paraaortic (38%), mediastinal (29%), and pelvic (17%) [3]. For axillary lymphadenopathy, incident data is not available. Nodal involvement rate was maximum in serous tumors (36.7%) followed by clear cell carcinoma (16.9%), endometroid tumors (15.6%), and mucinous tumors (7.7%) in order of decreasing frequencies [3].

The most common ovarian malignancy metastasizing to the axillary lymph nodes is serous carcinoma. Many of these patients can also have concurrent breast cancer. Whenever a patient with history of cancer has axillary lymphadenopathy, primary breast cancer should always be ruled out as a cause of metastasis. This is important because the treatments of these two entities are different. Cases with ovarian carcinoma which initially presented as axillary lymphadenopathy are also noted in the literature [3, 4]. In our patient we ruled out primary breast cancer by mammogram and breast sonogram. Cytopathology of breast and ovarian cancers can be very similar and can cause diagnostic dilemma in axillary lymph node metastasis [3]. Metastasis of ovarian cancer to breast is uncommon and is usually from serous ovarian carcinoma in 77.7% of cases, based on histology [5]. 

**Figure 3:**
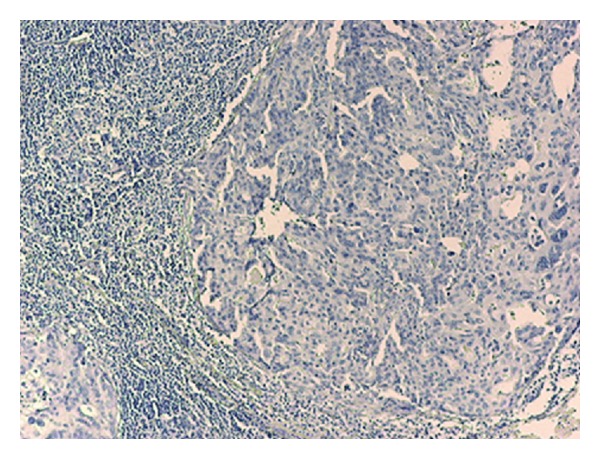
CK 20 negative axillary lymph node.

Immunohistochemical markers may aid in determining the origin of ALN metastases. Cytokeratin 7 (CK7) and cytokeratin 20 (CK20) are low-molecular-weight cytokeratins. Our patient had CK7^+^/CK20^−^ showing that this metastasis was epithelial in origin most probably coming either from ovary or breast [6] (Figure 3).

**Figure 4:**
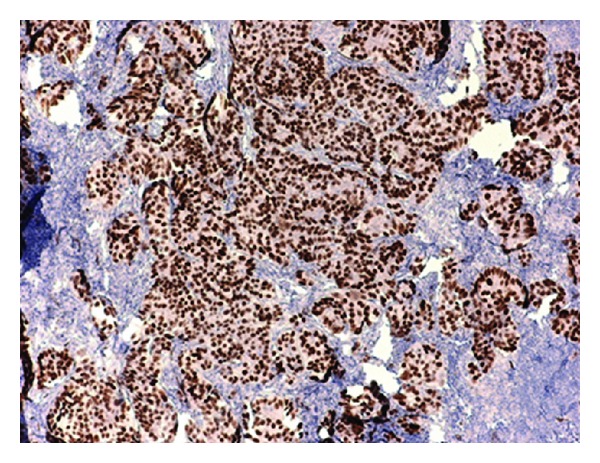
WT-1 positivity in axillary lymph node.

CA-125 levels can be measured in patient with axillary lymphadenopathy if origin of the metastases is obscure. CA-125 level is rarely elevated in breast cancer patients. It usually falls after cytoreductive surgery and chemotherapy. In our patient, CA-125 levels rose from 4.4 U/mL to 104 U/mL which indicated that this ALN was most likely related to this patient's previous history of ovarian cancer [7]. Wilms Tumor gene (WT-1) which is a tumor suppressor gene located on chromosome 11 at p13 found to be positive in 94.7% of patient with ovarian carcinoma was also positive in our patient [8] (Figure 4).

**Figure 5:**
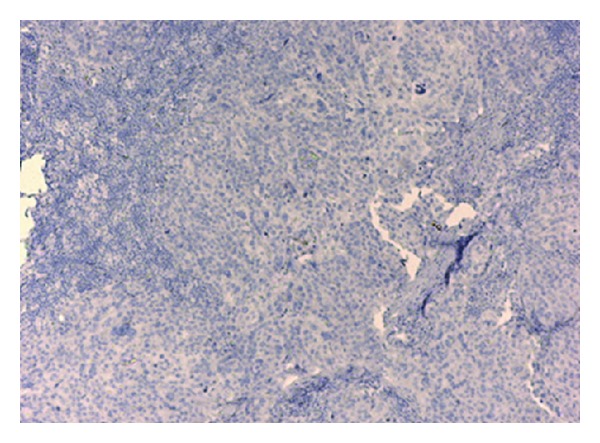
GCDFP-15 negative axillary lymph node.

Gross cystic disease fluid protein-15 (GCDFP-15) has been shown to be very sensitive and specific for breast cancer. Monteagudo et al. showed the value of GCDFP-15 in distinguishing metastatic breast carcinoma from poorly differentiated ovarian carcinoma. They used avidin-biotin-peroxidase technique to show that about 71% of metastatic breast cancers were positive for GCDFP-15 but none of the primary ovarian cancers were noted to be positive [9]. Our patient was negative for GCDFP-15 showing that the ALN in our patient was not arising from breast cancer (Figure 5).

In our patient we thought that ALN was a metastasis from breast cancer but it turned out to be metastasis from ovarian cancer and CA-125 level returned to normal range after resection of ALN. This patient has remained in remission from the past 3 yrs and is being observed in a routine manner. The authors suggest that, in patients with history of ovarian cancer who present with axillary mass, every effort should be made to have an accurate diagnosis since this has a great impact on treatment. We also emphasize the importance of physical examination in the patients with history of cancers.

## Figures and Tables

**Table 1:** CA-125 level trend in the patient.

Time frame	CA-125 Level (U/mL)
At the time of diagnosis of ovarian cancer	10302.7
After surgery	5328.1
After chemotherapy	4.4
After appearance of axillary lymph node	104
After excisional biopsy of lymph node	5
